# Protein Z: A putative novel biomarker for early detection of ovarian cancer

**DOI:** 10.1002/ijc.30020

**Published:** 2016-02-19

**Authors:** Matthew R. Russell, Michael J. Walker, Andrew J. K. Williamson, Aleksandra Gentry‐Maharaj, Andy Ryan, Jatinderpal Kalsi, Steven Skates, Alfonsina D'Amato, Caroline Dive, Maria Pernemalm, Phillip C. Humphryes, Evangelia‐Ourania Fourkala, Anthony D. Whetton, Usha Menon, Ian Jacobs, Robert L.J. Graham

**Affiliations:** ^1^ Stoller Biomarker Discovery Centre and Pathology NodeInstitute of Cancer Sciences, Faculty of Medical and Human Sciences, University of ManchesterManchesterUnited Kingdom; ^2^ Gynaecological Cancer Research Centre, Women's Cancer, Institute for Women's Health, University College LondonLondonUnited Kingdom; ^3^ Biostatistics Centre, MA General HospitalBostonMA; ^4^ Clinical and Experimental Pharmacology GroupCancer Research UK Manchester Institute, University of ManchesterManchesterUnited Kingdom; ^5^ SciLifeLab, Department of Oncology and PathologyKarolinska InstitutetTomtebodavägen 23, 171 65SolnaSweden; ^6^ University of New South WalesSydneyAustralia

**Keywords:** ovarian cancer, UKCTOCS, early detection, proteomics, SWATH

## Abstract

Ovarian cancer (OC) has the highest mortality of all gynaecological cancers. Early diagnosis offers an approach to achieving better outcomes. We conducted a blinded‐evaluation of prospectively collected preclinical serum from participants in the multimodal group of the United Kingdom Collaborative Trial of Ovarian Cancer Screening. Using isobaric tags (iTRAQ) we identified 90 proteins differentially expressed between OC cases and controls. A second targeted mass spectrometry analysis of twenty of these candidates identified Protein Z as a potential early detection biomarker for OC. This was further validated by ELISA analysis in 482 serial serum samples, from 80 individuals, 49 OC cases and 31 controls, spanning up to 7 years prior to diagnosis. Protein Z was significantly down‐regulated up to 2 years pre‐diagnosis (*p* = 0.000000411) in 8 of 19 Type I patients whilst in 5 Type II individuals, it was significantly up‐regulated up to 4 years before diagnosis (*p* = 0.01). ROC curve analysis for CA‐125 and CA‐125 combined with Protein Z showed a statistically significant (*p*= 0.00033) increase in the AUC from 77 to 81% for Type I and a statistically significant (*p*= 0.00003) increase in the AUC from 76 to 82% for Type II. Protein Z is a novel independent early detection biomarker for Type I and Type II ovarian cancer; which can discriminate between both types. Protein Z also adds to CA‐125 and potentially the Risk of Ovarian Cancer algorithm in the detection of both subtypes.

## Abstract 2

**What's new?**

New biomarkers for the early detection of ovarian cancer (OC) are urgently needed. In this blinded, prospective study, the authors identified Protein Z as a novel early marker for Type I and Type II OC that can also discriminate between the two types. When Protein Z levels were combined with CA‐125, results for early detection were even better. They may also enhance the Risk of Ovarian Cancer algorithm. These findings should help researchers develop more sensitive and specific screening panels for early‐stage OC.

**Abbreviations**
iRT: indexed retention time;
iTRAQ: isobaric tagging for relative and absolute quantitation
LC: Liquid Chromatography
MS: Mass Spectrometry
OC: ovarian cancer
ROCA: Risk of Ovarian Cancer Algorithm
SWATH: Sequential Window Acquisition of all Theoretical fragment ion spectra
UKCTOCS: United Kingdom Collaborative Trial of Ovarian Cancer Screening

Ovarian cancers (OC) can be classified into Type I (more indolent tumors lacking mutations in TP53) and Type II (aggressive cancers displaying TP53 mutations in >80% of cases).^1^

Mortality attributed to OC exceeds that of any gynaecological cancer,^2^, ^3^ with 4,272 deaths in the UK in 2011,^4^ and an estimated 15,500 deaths in the USA in 2012.^5^ 5 year survival rates are >90% in patients with early stage disease compared with 5% in those with Stage IV disease.^6^ Identification and development of biomarkers capable of detecting OC early could improve outcomes.

Serum CA‐125 is the most widely used biomarker for OC.^7^ More recently HE4 has been identified as an OC marker that complements CA‐125.^8^ These markers have limitations of specificity for OC, elevation of serum CA‐125 can also occur in pregnancy, endometriosis and menstruation.^7^ HE4 is not elevated in these conditions but is elevated in other cancers.^9^ Previous efforts to supplement CA‐125 with a broader panel of biomarkers within the Prostate, Lung, Colorectal and Ovarian Cancer Study,^10^, ^11^ in samples collected at diagnosis, found putative panels were not discriminatory >6 months prior to diagnosis, perhaps because the biomarkers were associated with mature tumors.^10^, ^11^

The most promising strategy for early detection of OC is a combination of screening using CA‐125 (interpreted using ROCA) with transvaginal ultrasound scanning. In the United Kingdom Collaborative Trial of Ovarian Cancer Screening (UKCTOCS), this strategy demonstrated a high sensitivity and specificity of 89 and 99.8%, respectively,^12^ on the prevalence screen and 85.8% and 99.8%, respectively, at incidence screening.^13^ Research is on‐going to ascertain the effect of this screening on mortality and also on its cost effectiveness for population screening.

A major hurdle in identifying early detection cancer biomarkers is access to appropriate sample sets for discovery. In this study, we use prospectively collected specimens in a blinded evaluation design. Samples were collected from the UKCTOCS cohort of 202,638 participants representing the target population envisioned for application of the biomarker.^14^

The aim of this study was to identify potential early Stage OC biomarkers and to ascertain the capacity of these biomarkers to detect preclinical OC.

## Methods

### Study subjects and samples

UKCTOCS is a 13 center OC screening trial of 202,638 women and is described in more detail in Supplementary Methods (available online).

Forty nine women from the multimodal group of UKCTOCS diagnosed with invasive epithelial (9 Type I and 30 Type II) and borderline (10) OCs for whom serum samples were available <14 months and >32 months prior to diagnosis were identified. We grouped borderline tumors with Type I for analysis within this study.^15^, ^16^ The Type II cases were matched to 31 controls, with no family history of OC and no diagnosis of a cancer during follow‐up, on age, collection center and collection date. Multiple serial samples were available in these women—in total; the set consisted of 482 individual samples. The set was divided into: (*i*) a discovery set which included all women but only two samples from each woman, one at <14 months and the other at >32 months prior to diagnosis; and (*ii*) a validation set which included all serial samples from the same women spanning a 7 year period. Supporting Information Table 1 contains detailed information on all of these individual samples including which were used for iTRAQ, SWATH, ELISA and detected early by PROZ. It also contains information on the histology, morphology, grade, stage, age at which sample was taken and BMI

### Isobaric tagging for relative and absolute quantitation (iTRAQ) analysis

Serum samples from the discovery set above were pooled according to the two time points into six groups, four containing cancer samples with 25 individual samples in each and two control group samples (Supporting Information Table 2). Pooling increases the efficacy of the analysis and averages out individual variation.^17^ Serum was immunodepleted as described in Supporting Information Methods (available online).

### Sequential window acquisition of all theoretical fragment ion spectra (SWATH) analysis

At the end of 2013 all of the discovery samples underwent central review, this resulted in 6 Type I samples being reclassified‐ 3 as Type II and 3 as potential Type II; 2 controls subject had gone on to get other cancers and one Type II sample was queried.

From the discovery set new pools were prepared, (containing only those samples confidently classified by central review: 19 Type I subjects; the remaining 23 controls and 27 Type II subjects (25 Type II + 3 reclassified as Type II minus the queried Type II), according to the two time points, into six groups' four containing cancer samples and two control groups (Supporting Information Table 3).

### iTRAQ labeling and liquid chromatography mass spectrometry (LC‐MS)

Depleted/buffer exchanged serum protein was reduced and digested by the addition of trypsin (Promega), then labeled with 8plex iTRAQ reagents according to the manufacturer's instructions (AB Sciex). Labeled samples (Supporting Information Table 2) were fractionated and analyzed by MS as described previously.^18^

### SWATH mass spectrometry

SWATH analysis was carried out as outlined in Ref. 19 further details are described in Supporting Information Methods (available online).

### Spectral library creation

A spectral library is required to obtain quantitative protein information from SWATH maps. For the 20 proteins of interest (Supporting Information Table 4) peptides were synthesised (JPT peptide Technologies), mixed with indexed retention time (iRT) peptides (Biognosys AG) and loaded onto the LC‐MS system for analysis. Fragment ion spectra were combined with spectral information from an in house database to give the final spectral library.^20^

### Protein identification and quantitation

#### iTRAQ discovery

Mass spectrometry raw data files were converted to MGF format and searched, using MASCOT, against the Swissprot database (2012_11) with Taxonomy set to Human. MASCOT search parameters are described in Supporting Information Methods (available online).

### SWATH MS targeted data extraction using skyline

Spectral Libraries and raw .WIFF files from SWATH MS experiments were imported into Skyline,^21^ iRT peptides allowed retention time recalibration and normalization between samples.^22^ Protein abundances were estimated from ≥2 peptides per protein.

### ROCA classification

CA‐125 results within UKCTOCS were interpreted using the ROCA rather than a single cut‐off. All women randomised to the multimodal group of the trial underwent annual CA‐125 testing and based on the ROCA classification, they were either returned to annual screening (if their risk was normal) or triaged to repeat CA‐125 testing in 3 months (if intermediate risk) or repeat CA‐125 and transvaginal ultrasound in 6 weeks (if elevated risk).^12^, ^23^ Those with persistently elevated risk were sent for clinical assessment with a view to surgery. The trial protocol is detailed elsewhere.^12^, ^23^

### ELISA validation of protein Z

482 individual serum samples were run in triplicate using Protein Z ELISAs (Abcam, Cambridge, UK) according to manufacturer's instructions. Table 1 includes details of the set and Table 2 has information on the characteristics of the primary OCs. Serum CA‐125 level concentrations were available for all of the above samples as previously described.^12^

**Table 1:** Baseline characteristics of UKCTOCS participants used within this study

	Median (25th–75th centiles)							
	Control		Ovarian cancer					
			Overall		Type I		Type II	
	*n* = 31		*n* = 49		*n* = 19		*n* = 30	
Age (years) at randomisation	60.8 (58.4–65.8)		62.8 (58.7–67.3)		64.2 (58.9–69.9)		61.1 (58.7–65.5)	
Years since last period at randomisation	12.6 (6.6–18.2)		11.4 (5.7–18.2)		15.2 (8.1–22.6)		10.7 (4.0–16.1)	
Duration of HRT use in those who were on HRT at randomisation (yrs)	6.9 (5.8–11.7)		9.7 (4.8–13.0)		13.0 (10.7–13.9)		7.2 (3.3–11.6)	
Duration of OCP use (yrs) in those who had used it	10 (3–12)		6 (3–8)		5 (3–8)		6 (4–8)	
Miscarriages (pregnancies < 6mths)	0 (0–1)		0 (0–0)		0 (0–1)		0 (0–0)	
No. of children (pregnancies > 6mths)	2 (0–2)		2 (2–2)		2 (1–2)		2 (2–3)	
Height (cms)	162.6 (158.8–167.6)		162.6 (157.5–165.1)		162.6 (157.5–166.4)		162.6 (157.5–165.1)	
Weight (kg)	65.3 (62.6–74.0)		69.9 (62.6–78.9)		71.2 (66.7–79.2)		65.9 (61.7–75.8)	
	Number (%)							
	No.	%	No.	%	No.	%	No.	%
Ethnicity:								
White	30	96.8%	48	98.0%	18	94.7%	30	100.0%
Black	0	0.0%	0	0.0%	0	0.0%	0	0.0%
Asian	0	0.0%	0	0.0%	0	0.0%	0	0.0%
Other	1	3.2%	1	2.0%	1	5.3%	0	0.0%
Missing	0	0.0%	0	0.0%	0	0.0%	0	0.0%
Hysterectomy	1	3.2%	6	12.2%	2	10.5%	4	13.3%
Ever use of oral contraceptive pill	18	58.1%	21	42.9%	9	47.4%	12	40.0%
Use of HRT at recruitment	8	25.8%	9	18.4%	3	15.8%	6	20.0%
Personal history of cancer^a^	0	0.0%	2	4.1%	1	5.3%	1	3.3%
Personal history of breast cancer	0	0.0%	1	2.0%	1	5.3%	0	0.0%
Maternal history of ovarian cancer	0	0.0%	1	2.0%	0	0.0%	1	3.3%
Maternal history of breast cancer	4	12.9%	4	8.2%	1	5.3%	3	10.0%

a Includes those with personal history of breast cancer.

**Table 2:** Characteristics of the primary ovarian cancers

	No.			
Cancer type	Overall	Stage I	Stage II	Stage III
Type I	19	14	1	4
Borderline	10	10	0	0
Serous	6	6	0	0
Mucinous	2	2	0	0
Endometrioid	2	2	0	0
Invasive	9	4	1	4
Low grade endometrioid	5	3	1	1
Clear cell	3	0	0	3
Adenocarcinoma	1	1	0	0
Type II	30	7	8	15
High grade serous	23	5	6	12
High grade endometrioid	3	1	1	1
Carcinosarcoma	1	0	0	1
Adenocarcinoma	3	1	1	1

### Statistical analysis

iTRAQ Channels 119 and 121 were used to label a pool of all 150 samples from the initial discover phase which were used to assess technical variation within the work flow, proteins identified with ≥ 2 peptides with a significance threshold of *p* < 0.05 and having relative ratios outside the 95% confidence intervals were identified as having significant fold changes.^24^

Wilcoxon signed rank test, Loess Linear regression, nonlinear modeling, support vector machine learning for ROC curve construction and MS Stats analysis were all carried out using the R environment for statistical analysis. All *p* values are two tailed and those <0.05 were considered statistically significant.

## Results

### Subjects

Baseline characteristics for cases and controls are shown in Table 1. The mean age was 62.8 in cases and 60.8 in controls. In the Type I cancers, 10 were borderline and of the remaining 9, five were low grade endometrioid, one was adenocarcinoma and three were clear cell (Table 2).

#### Proteomic analysis of OC samples

To study global protein expression for the detection of early biomarkers of OC, pooled samples were iTRAQ labeled (Supporting Information Fig. 1a) and analyzed at <14, >32 months to diagnosis, (Supporting Information Table 2). Samples were run in triplicate with ninety proteins displaying significant differential expression between cases and controls (Supporting Information Table 4 contains detailed information on all the proteins, including if they have previously been identified as potential ovarian cancer biomarkers).

**Figure 1:**
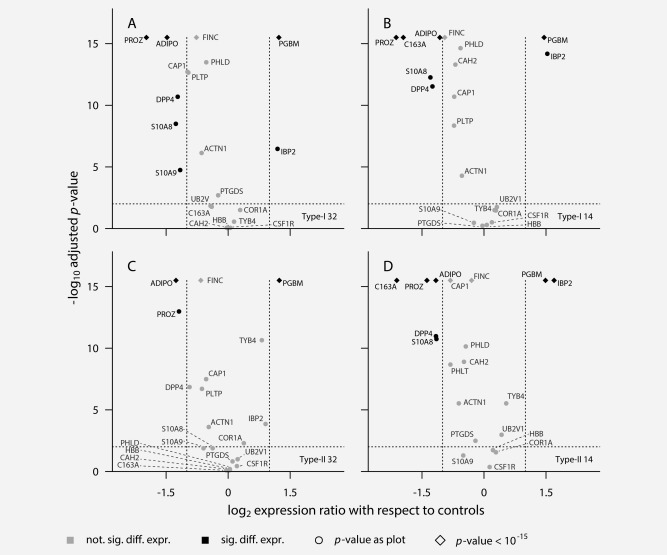
Volcano plots comparing differential expression of the 20 putative early detection biomarkers versus control levels across the ovarian cancer sub types and time points under investigation. The horizontal dotted line is the 95% confidence interval. Proteins to the right of the right hand vertical dotted line are at least twofold increased in expression compared with control. Proteins to the left of the left hand vertical dotted line are at least twofold decreased in expression compared with control. (*a*) Expression of proteins for Type I ovarian cancer at the farthest point from diagnosis (>32 months). (*b*) Expression of proteins for Type I ovarian cancer at the earlier point from diagnosis (<14 months). (*c*) Expression of proteins for Type II ovarian cancer at the farthest point from diagnosis (>32 months). (*d*) Expression of proteins for Type II ovarian cancer at the earlier point from diagnosis (<14 months).

Spectral libraries were created for twenty of these proteins (Supporting Information Table 4) which were further verified using an orthogonal mass spectrometry technique, SWATH MS (Supporting Information Fig. 1b). New pools were generated for the Type I and Type II patients along with matched controls at <14/>32 months prior to diagnosis (Supporting Information Table 3). SWATH maps were created for each, in triplicate, interrogated using targeted data extraction *via* the spectral libraries above^19^ and quantitative information was extracted using Skyline^21^ and MS Stats.^25^ Volcano plots were then constructed (Fig. 1) to identify putative biomarkers that were most discriminatory between OC cases and control subjects enabling the identification of markers that may be of utility in early detection.

### Validation of early detection biomarkers

Three proteins discriminated between OC cases and control subjects at all time points identifying them as potential early screening biomarkers. These were PGBM; basement membrane‐specific heparin sulfate proteoglycan core protein, ADIPO; Adiponectin and PROZ; Vitamin K dependant protein Z (Protein Z). PGBM is a large (>400 kDa) multidomain protein which is abundantly expressed in basement membranes and the ECM of normal blood vessels and tissues where it maintains the function of the endothelial barrier.^26^ Adiponectin is an adipocyte‐derived cytokine involved in a number of metabolic processes, including glucose regulation and fatty acid oxidation. It has anti‐inflammatory and anti‐atherogenic effects and plays a protective role in experimental models of vascular injury.^27^ Protein Z is an anticoagulant that accelerates the inhibitory effect of PZ‐dependent protease inhibitor on coagulation factor Xa.^28^

Protein Z was the only one of these three not to have been associated with OC previously, as a potential novel biomarker it was selected for further validation by commercially available assays. PROZ ELISAs were run in triplicate, on 482 individual serum samples representing 80 individuals; 30 Type II OC women; 31 controls and 19 Type I women, all with serial serum samples spanning up to 7 years prior to diagnosis. Table 1 contains information on all samples investigated. Results for women with Type I and Type II OC versus healthy control subjects were analyzed separately and compared with each individuals CA‐125 level and ROCA classification.

### Protein Z identified as an early marker for Type I OC

**Figure 2:**
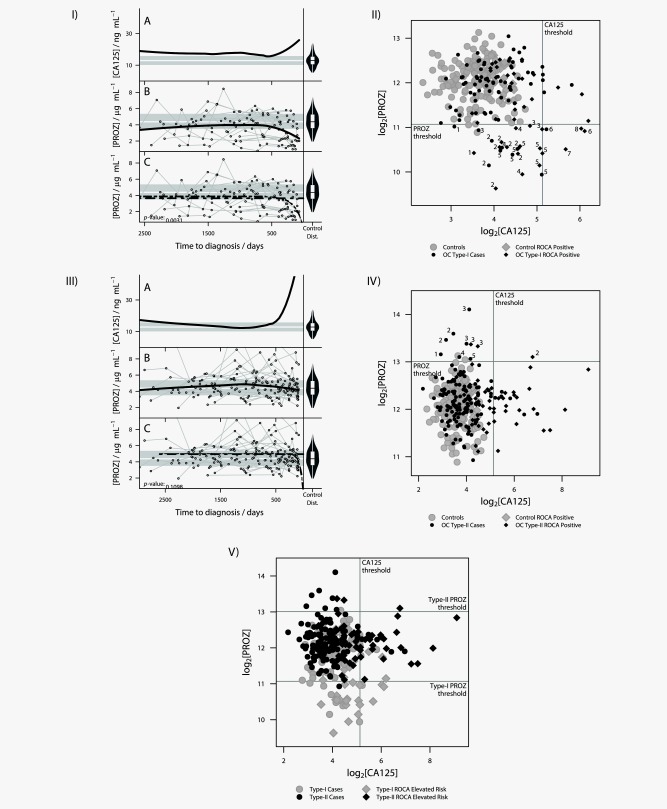
Analysis of Type I and Type II ovarian cancer patients within the validation cohort. (*I*) Analysis of Type I ovarian cancer patient validation cohort (*Ia*) Loess linear regression analysis of trends in CA‐125 levels in Type I subjects. On the right hand side is a violin plot of the distribution of the CA‐125 levels in control subjects. The gray horizontal bars represent the interquartile range of the controls with the white horizontal line in between being the median level of CA‐125 in the control population. The thick black line represents the trend in the data identified by the Loess analysis. (*Ib*) Loess linear regression analysis of Protein Z levels in Type I subjects. On the right hand side is a violin plot of the distribution of the Protein Z levels in control subjects. Ovarian cancer patient Protein Z levels are represented by circles and individual patient levels over time are shown by connected circles. The gray horizontal bars represent the interquartile range of the controls with the white horizontal line in between being the median level of Protein Z in the control population. The thick black line represents the trend in the data identified by the Loess analysis. (*ic*) Nonlinear modelling of the data from (*Ib*). Two models were tested, flat expression represented by the horizontal dashed line and exponential decay represented by the second dashed line. (*II*) Comparison of the discriminatory power of CA‐125 versus Protein Z for Type I ovarian cancer cases. The vertical line represents the CA‐125 threshold above which women would be sent to a gynecological oncologist (35U ml^−1^). The horizontal line represents the Protein Z threshold which was selected by taking the 1^st^ percentile of all control samples, representing an empirical estimate of a 1% FDR. The numbers beside cases ascertains which individuals each point relates to. (*III*) Analysis of Type II ovarian cancer patient validation cohort. (*IIIa*) Loess linear regression analysis of trends in CA‐125 levels in all subjects. On the right hand side is a violin plot of the distribution of the CA‐125 levels in control subjects. The gray horizontal bars represent the interquartile range of the controls with the white horizontal line in between being the median level of CA‐125 in the control population. The thick black line represents the trend in the data identified by the Loess analysis. (*IIIb*) Loess linear regression analysis of Protein Z levels in all subjects. On the right hand side is a violin plot of the distribution of the Protein Z levels in control subjects. Ovarian cancer patient Protein Z levels are represented by circles and individual patient levels over time are shown by connected circles. The gray horizontal bars represent the interquartile range of the controls with the white horizontal line in between being the median level of Protein Z in the control population. The thick black line represents the trend in the data identified by the Loess analysis. (*IIIc*) Nonlinear modelling of the data from (*IIIb*). Two models were tested flat expression represented by the horizontal dashed line and exponential decay represented by the second dashed line. (*IV*) Comparison of the discriminatory power of CA‐125 versus Protein Z for Type II ovarian cancer cases. The vertical line represents the CA‐125 threshold above which women would be sent to a gynecological oncologist (35 U ml^−1^). The horizontal line represents the Protein Z threshold which was selected by taking the 99th percentile, of all control samples, representing an empirical estimate of a 1% FDR.The numbers beside cases ascertains which individuals each point relates to. (*V*) Comparison of Protein Z versus CA‐125 levels for Type I OC and Type II OC. The vertical line represents the CA‐125 threshold above which women would be sent to a gynecological oncologist (35 U ml^−1^). The lower PROZ threshold applied to Type‐I OC was selected by taking the 1st percentile and the upper threshold applied to Type‐II OC the 99th percentile, of all control samples. This represents an empirical estimate of a 1% FDR in each case.

Loess linear regression analysis of CA‐125 levels for Type I women (Fig. 2Ia) were compared with their Protein Z levels (Fig. 2Ib). It was found that as CA‐125 levels rise toward diagnosis (breakpoint 420 days (∼ 60 weeks) prediagnosis), there was a mirrored corresponding decrease in the level of Protein Z.

The Protein Z Loess line tracks below the median value for the control population dropping more dramatically below the median value at less than 500 days to diagnosis. Some individuals' Protein Z values track below the quartile range during the whole time course and after 500 days to diagnosis several individuals were below the lowest values identified in the control population (Fig. 2Ib) .

To ascertain if this was a real decrease we assessed two nonlinear models for Protein Z (Fig. 2Ic): level expression as the optimum method for defining the data; decay in expression best describing the data. The latter, best defined the data with a *p* values = 0.0031 demonstrating that the decrease in expression is real. The inflection point on the Loess plots for CA‐125 and Protein Z occurs at 420 days (∼60 weeks) prior to diagnosis. Protein Z levels were placed into 60 weeks bins, covering the full 7 year timespan and compared with levels in the control subjects. There was a significant decrease in the Protein Z levels, within the 60 weeks to diagnosis bin, compared with control levels (*p* = 0.000000411).

### Protein Z complements CA‐125 and ROCA in detection of Type I OC

We next compared the capability of CA‐125 and Protein Z to discriminate between Type I OC patients and healthy controls (Fig. 2II). Protein Z levels significantly decreased in comparison to controls in eight of the 19 cases (patients 1–8, Supporting Information Table 5); with ∼25% of the Type I data points falling below the Protein Z threshold (Fig. 2II); identifying both invasive and borderline cases at a median of 385.5 days (∼1 year) prediagnosis, all with Stage I/II disease.

In patients 1–5 CA‐125 levels did not rise sufficiently for them to be identified using CA‐125 alone. The eight women identified below the Protein Z threshold were ROCA screen positive (intermediate/elevated risk). For patients 1–5 Protein Z identified them substantially earlier than ROCA (Supporting Information Table 6).

ROC curves were constructed for CA‐125, Protein Z and for CA‐125 and Protein Z combined (Supporting Information Fig. 2). The combined panel showed a significant (*p* = 0.00033) increase in the AUC from 77% for CA‐125 alone and 73% for Protein Z alone to 81%.

### Protein Z identified as a marker for Type II OC

Loess linear regression analysis of CA‐125 levels for Type II women (Fig. 2IIIa) were compared with their Protein Z levels (Fig. 2IIIb). The Protein Z Loess line tracks on the median line for the controls apart from between 1,500 and 500 days prediagnosis where there is a noticeable rise above then return to control median values (Fig. 2IIIb).

To ascertain if the trend seen for the Type I patients was specific, we tested the nonlinear models used in the Type I analysis on the Type II dataset, no significant trend was demonstrated (Fig. 2IIIc). As with the Type I dataset Protein Z levels for the Type II cases were placed into 60 weeks bins and compared with levels in the control subjects. There was a less marked but significant increase in the Protein Z levels in the 120–180 weeks prediagnosis bin compared with controls (*p* = 0.0105183).

### Protein Z complements CA‐125 and ROCA in detection of Type II OC

We compared the capability of CA‐125 and Protein Z to discriminate between Type II OC patients and healthy controls (Fig. 2IV). Protein Z levels were seen to significantly increase in five of the 30 Type II cases (Patients 1–5, Supporting Information Table 7); with ∼5% of the Type II data points rising above the Protein Z threshold (Fig. 2IV); cases were identified at a median of 1,083 days (∼3 years) prediagnosis, with Stage I/II/III disease.

Four patients' CA‐125 levels did not rise sufficiently for them to be identified using this screening method alone. All five women identified above the Protein Z threshold were also ROCA screen positive. For these women Protein Z identified them substantially earlier than ROCA (Supporting Information Table 6). ROC curves were constructed for CA‐125, Protein Z and for CA‐125 and Protein Z combined (Supporting Information Fig. 2). The combined panel showed a significant (*p* = 0.00003) increase in the AUC from 76% for CA‐125 alone and 54% for Protein Z alone to 82%.

### Protein Z levels are mutually exclusive for Type I and Type II OC

We compared whether Protein Z levels could discriminate between Type I and Type II cases (Fig. 2V). Protein Z is mutually exclusive for Type I and II, only one of the Type II data points breaches the low Protein Z Type I threshold and none of the Type I data points breach the Type II threshold. More detailed features from Figure 2V showing individual patient samples and the samples proximity to diagnosis can be seen in Supporting Information Figure 3.

## Discussion

We carried out a blinded evaluation using prospectively collected samples from UKCTOCS^14^ which contains serum samples from >200,000 women in the UK, with serial samples from >50,000 women, spanning a 10 year period. This enabled us to evaluate putative biomarkers for detection of preclinical disease.

We employed two unbiased, complementary proteomic approaches to profile matched serial case control sets of serum samples. This identified Protein Z, as a discriminator between OC cases and controls with potential as an early screening marker. Protein Z enhances by >1,000 fold the inhibition of factor Xa by Protein Z dependant protease inhibitor.^29^ Deregulation of the haemostatic system is often seen in malignancy with localised activation of coagulation at tumor sites facilitating cancer progression.^30^ Activation of factor X is critical in the coagulation pathway and it has been shown to play an important role in the deregulation of haemostasis seen in malignancy.^31^ Changes in expression of Protein Z in OC may be related to its role within this process.

Comparison of CA‐125 and Protein Z in their ability to identify Type I OC showed that Protein Z expression identified eight of the 19 Type I OC cases, both invasive and borderline, all with Stage I/II disease. Five of these women showed no significant elevation in their CA‐125 levels and would therefore not have been diagnosed by analysis of CA‐125 alone. All eight women were ROCA positive,^32^ five were identified substantially earlier by Protein Z than by ROCA, adding weight to Protein Z as an early detection marker that could add to and complement current prediction models for the early detection of Type I OC. Protein Z down regulation is specific for the early detection of Type I OC and not a marker of general malignant progression in OC, as it is not significantly down regulated within Type II OC cases.

Analysis of CA‐125 and Protein Z in their ability to identify Type II OC showed that significant up‐regulation of Protein Z allowed the identification of five Type II women. Four of these women showed no significant elevation in their CA‐125 levels and would therefore not have been diagnosed by the analysis of CA‐125 alone. In addition, of the 15 late stage cases present we found that Protein Z only showed elevated levels in two, we are uncertain why this is the case. The five individuals identified by an increase in Protein Z expression were ROCA positive; all five women were identified substantially earlier by Protein Z suggesting that in certain Type II cases it may add to the risk algorithm.

While Protein Z out performed ROCA with a greater lead time in five of the Type I women it identified and 5 of the Type II women; ROCA is still superior as an estimation tool for the risk of a women having OC at the time of sampling as it identified all of the OC cases within the study.

Current algorithms both for OC screening (ROCA^32^) and differential diagnosis of an adnexal mass in symptomatic patients (OVA1^33^ or ROMA^8^, ^34^) give significant weighting to CA‐125 levels. We constructed ROC curves for CA‐125 and for CA‐125 and Protein Z combined. The combined panel showed a significant (*p* = 0.00033) increase in the AUC from 77% for CA‐125 alone to 81% for Type I and a significant (*p* = 0.00003) increase in the AUC from 76% for CA‐125 alone to 82% for Type II OC demonstrating that Protein Z adds to CA‐125 for the detection of Type I and Type II OC.

Unlike most biomarkers which rise toward diagnosis, Protein Z levels were significantly down regulated in Type I OC compared with those of controls with a median case identification time of 385 days (∼1 year) prior to diagnosis. An important consideration here is lead time as the women enrolled in UKCTOCS who were detected by screening were diagnosed 1–2 years on average before a clinical diagnosis would have been made. These results suggest that Protein Z has the ability to detect OC Type I cases with considerable lead time.

Interestingly Protein Z rose significantly in Type II cases compared with controls with a median case identification of 1,083 days (∼3 years) prediagnosis, taking into account lead time due to study inclusion, for those cases identified that gives a potential lead time of as much as 4 years.

We have established the power of UKCTOCS and the strength of advanced mass spectrometry/clinical proteomics in the identification of biomarkers for Type I and Type II OC. We have shown that Protein Z is an early detection biomarker for both subtypes, with the ability to clearly discriminate between the two. Protein Z appears to be a better marker for Type I as it identified a greater proportion of cases (eight out of 19) than Type II (five out of 30) and captured more of the available Type I data points (∼25%) compared with Type II (∼5%). The identification of Protein Z as a potential early detection biomarker for Type I and Type II OC is significant.

Further validation needs to be carried out before the clinical utility of Protein Z could be implemented, such studies should include samples from patients with benign tumors and other unrelated diseases. Once this is completed, one can envision potential clinical utility of Protein Z within two areas. The first is in its incorporation into current screening protocols, for example, where Protein Z levels could be measured at the same time as CA‐125 levels and both could be assessed through the ovarian cancer risk estimation algorithm ROCA.

The second could be the incorporation of Protein Z levels within current algorithms for differential diagnosis of an adnexal mass in symptomatic patients; where its addition to the OVA1^33^ or ROMA^8^, ^34^ could aid in triage of high risk symptomatic patients presenting at a clinic. This current work moves us toward both of these possibilities and, with the addition of other markers, potentially closer to the production of discriminatory panels for the identification of early stage OC.

## Supporting information

Supporting Information

Supporting Information Figure 1

Supporting Information Figure 2

Supporting Information Figure 3

Supporting Information Table 1

Supporting Information Table 2

Supporting Information Table 3

Supporting Information Table 4

Supporting Information Table 5

Supporting Information Table 6

Supporting Information Table 7
