# Supplementary material for: Protein Z: A putative novel biomarker for early detection of ovarian cancer
Source: Int J Cancer. 2016 Feb 19;138(12):2984–92. doi: 10.1002/ijc.30020 (PMC4840324; doi:10.1002/ijc.30020)
Supplement: Supplementary file 1 — Supporting Information [file IJC-138-2984-s001.doc]

Supplementary Methods:

**Study, subjects and samples**-

UKCTOCS (International Standard Randomised Controlled Trial, number ISRCTN22488978; ClinicalTrials.gov NCT00058032) was approved by the UK North West Multicentre Research Ethics Committees (North West MREC 00/8/34). Trial design, including eligibility criteria and details of recruitment have been described in detail elsewhere . All women provided written informed consent for use of their samples in secondary studies. The current biomarker discovery study was approved by the Joint UCL/UCLH Committees on the Ethics of Human Research (Committee A) (Reference No. 05/Q0505/57).

UKCTOCS is a 13 centre ovarian cancer screening trial of 202,638 women randomised to annual screening with CA125 interpreted using ROCA with transvaginal ultrasound (TVS) as a second line test (multimodal group, n=50,640); TVS (n=50,639) or control group (n=101,359). Between 2001-2011, the women in the multimodal group donated serum samples at their annual screening appointment. The blood samples were taken at the trial centres using gel tubes (Greiner Bio-One; 455071, 8ml Gel Separation Serum Tubes) and transported overnight at ambient temperature to the central laboratory. All the samples received >56 hours after venepuncture were discarded and repeat samples were requested. The blood samples were centrifuged at 4,000 rpm for 10 minutes and the serum separated. Excess serum was stored in aliquots. Serum CA125 levels were determined by electrochemiluminescence sandwich immunoassay on an Elecsys 2010 (Roche Diagnostics) employing two monoclonal antibodies (OC125 and M11) .

All participants were followed up with the Health and Social Care Information Centre (HSCIC) for England and Wales and with the Central Service Agency and Cancer Registry in Northern Ireland for cancer registration and deaths. In women identified to have ovarian cancer, case notes were reviewed by an Outcomes Review Committee who assigned the final diagnosis, stage, morphology of any cancer and where possible, classified iEOC into Type I (low-grade serous, low-grade endometrioid, mucinous and clear cell cancers) or Type II (high-grade serous, high-grade endometrioid, carcinosarcomas and undifferentiated carcinoma) cancers .

**Serum Immunodepletion**-

Serum was centrifuged at 4^o^C (15700 x g for 5 min) to remove particulates. Supernatant (40 µL) was immunodepleted using a 4.6 x 100 mm multiple affinity removal (Human 14) liquid chromatography column, following the manufacturers protocol (Agilent, USA) using an Agilent 1260 HPLC system (Agilent, USA). Depleted serum samples were then concentrated, buffer exchanged and the protein concentration measured using the Bradford method (Sigma-Aldrich, USA).

**iTRAQ labelling and liquid chromatography mass spectrometry (LC-MS)**

iTRAQ labelled samples were run on either a TripleTOF 5600 or an Orbitrap Velos. Raw mass spectrometry files were converted to MGF and processed using the following MASCOT search parameters: Quantitation was set to iTRAQ 8-plex; enzyme was set to trypsin allowing for 1 missed cleavage; fixed modifications were set to Methylthio (C),iTRAQ8plex (N-term),iTRAQ8plex (K); variable modifications were set to Acetyl (Protein N-term),Deamidated (NQ),Oxidation (HW),Oxidation (M), Phospho (ST),Phospho (Y),iTRAQ8plex (Y); peptide mass tolerance was set to 20ppm and fragment mass tolerance to 0.3 Da. The decoy database option was enabled with an FDR of 1%.

**Sequential Window Acquisition of all Theoretical fragment ion mass spectrometry –SWATH-MS**

Depleted serum (10 ug) was reduced, alkylated and digested overnight at 37^o^C with sequencing grade trypsin (Promega, USA). Samples were dried at 45^o^C using a miVac DNA Concentrator, reconstituted and indexed retention time peptides ( iRT) (Biognosys AG) (used as retention time anchor points and for normalisation) were added at a ratio of 1:30 v/v . 0.5ug of sample was loaded onto the LC-MS system and analysed over 180 minutes.

Mass spectrometry raw data files can be freely accessed at <http://www.iprox.org/index> project id IPX00072300.

1. Menon U, Gentry-Maharaj A, Hallett R*, et al.* Sensitivity and specificity of multimodal and ultrasound screening for ovarian cancer, and stage distribution of detected cancers: results of the prevalence screen of the UK Collaborative Trial of Ovarian Cancer Screening (UKCTOCS). The Lancet Oncology 2009;10(4):327-340.

2. Menon U, Gentry-Maharaj A, Ryan A*, et al. Recruitment to multicentre trials—lessons from UKCTOCS: descriptive study*; 2008.

3. Menon U, Ryan A, Kalsi J*, et al.* Risk Algorithm Using Serial Biomarker Measurements Doubles the Number of Screen-Detected Cancers Compared With a Single-Threshold Rule in the United Kingdom Collaborative Trial of Ovarian Cancer Screening. Journal of Clinical Oncology 2015.

4. Liu Y, Hüttenhain R, Surinova S*, et al.* Quantitative measurements of N-linked glycoproteins in human plasma by SWATH-MS. PROTEOMICS 2013;13(8):1247-1256.

Supplementary figure legends

Supplementary Figure 1. Overview of clinical proteomics pipeline: (A) Quantitative iTRAQ analysis: (i) Protein abundance in serum is in excess of 10 orders of magnitude. We established an immuno-depletion protocol for serum samples using the Mars 14 multiple affinity removal system to allow deeper proteomic mining. (ii) Following immuno-depletion proteins are digested with trypsin, producing peptides containing free amine groups. (iii) Peptides from up to 8 samples are labelled with individual isobaric tags (iTRAQ with individual reporter groups 113-121) for analysis and comparison. (iv &v) Samples are pooled and fractionated using high pH reverse phase separation. (vi) Fractions are analysed using data dependant analysis on a mass spectrometer. Here the peptide is identified and the reporter group is released with measurement of the area of each reporter giving a measurement of the abundance of that peptide present in each sample. (vii) Data for peptides from the same protein is then integrated to give values on protein level changes with full statistical significance provided.

(B)Targeted SWATH analysis: (i) Production of spectral libraries: proteotypic peptides were selected, synthesised and mixed with indexed retention time (iRT) peptides. These were analysed on a mass spectrometer to produce a spectral library, which contains all of the information on fragment ion signals, their relative intensities, chromatographic concurrence and other data used to mine the SWATH maps for signals that uniquely identify the target protein. (ii) After immunodepletion and protein digestion, samples were run on a mass spectrometer in data independent acquisition mode to produce SWATH maps. (iii) Protein identification and quantitation was carried out using targeted data extraction from the SWATH maps based on the spectral library produced in (i). Fragment ion chromatograms for each peptide of interest were extracted from the SWATH MS maps and their abundances compared between the different conditions for expressional analysis. (iv) bioinformatics analysis using Skyline and MS Stats allowed the quantitation and statistical analysis of the proteins in all sample sets under investigation.

Supplemental Figure 2. ROC curve analysis of sensitivity vs specificity, PPV and NPV for CA-125 and CA-125 plus Protein Z for (I) Type I OC and (II) Type II OC and for Protein Z alone (III) Type I OC and (IV) Type II OC.

Supplementary figure 3. More detailed information on individuals represented in figure 2V. Comparison of Protein Z vs CA-125 levels for Type I OC and Type II OC. The vertical line represents the CA-125 threshold above which women would be sent to a gynecological oncologist (35U ml^-1^). The lower PROZ threshold applied to Type-I OC was selected by taking the 1^st^ percentile and the upper threshold applied to Type-II OC the 99^th^ percentile, of all control samples. This represents an empirical estimate of a 1% FDR in each case. The numbers beside cases ascertains which individuals each point relates to. As the shapes get larger they are closer to diagnosis with smaller shapes being farther from diagnosis.

Supplemental table 1

Detailed information on all individual serum samples including which were used for iTRAQ , SWATH, ELISA and detected early by PROZ and also containing information on histology, morphology, grade, stage, age at which sample was taken and BMI.

Supplemental table 2

Summary data of the serum samples from the multimodal arm of UKCTOCS used in the iTRAQ quantitative analysis.

(tDx- time to diagnosis. Standard deviation of values are represented in brackets)

Supplemental

Supplemental table 3

Summary data of the serum samples from the multimodal arm of UKCTOCS used in the SWATH targeted analysis.

(tDx- time to diagnosis. Standard deviation of values are represented in brackets)

Supplemental table 4

Contains information on all 90 iTRAQ differentially expressed proteins, with information on their molecular function and enzyme and pathway annotation and whether it has been identified as being involved in any diseases, from the Uniprot database. The table highlights where the proteins have been identified as OC biomarkers previously by cross reference with the review by Lokshin and Nolen where they collate all known putative ovarian cancer biomarkers within the literature. In addition the table highlights whether the proteins were potential Type I, Type II or Type I & Type II markers, as well as identifying the 20 proteins that were selected for SWATH verification.

Supplemental table 5

Information on the individual patients’ histology, morphology, stage, grade and time to diagnosis for the Protein Z identified Type I OC cases.

Supplemental table 6

Table showing lead time in days provided by Protein Z, when subjects first cross the threshold, for the 5 Type I and 5 Type II OC subjects compared to when ROCA first identified these individuals as intermediate or elevated risk.

Supplemental table 7

Information on the individual patients’ histology, morphology, stage, grade and time to diagnosis for the Protein Z identified Type II OC cases.
