# Supplementary figures and images for: Protein Z: A putative novel biomarker for early detection of ovarian cancer
Source: Int J Cancer. 2016 Feb 19;138(12):2984–92. doi: 10.1002/ijc.30020 (PMC4840324; doi:10.1002/ijc.30020)

A

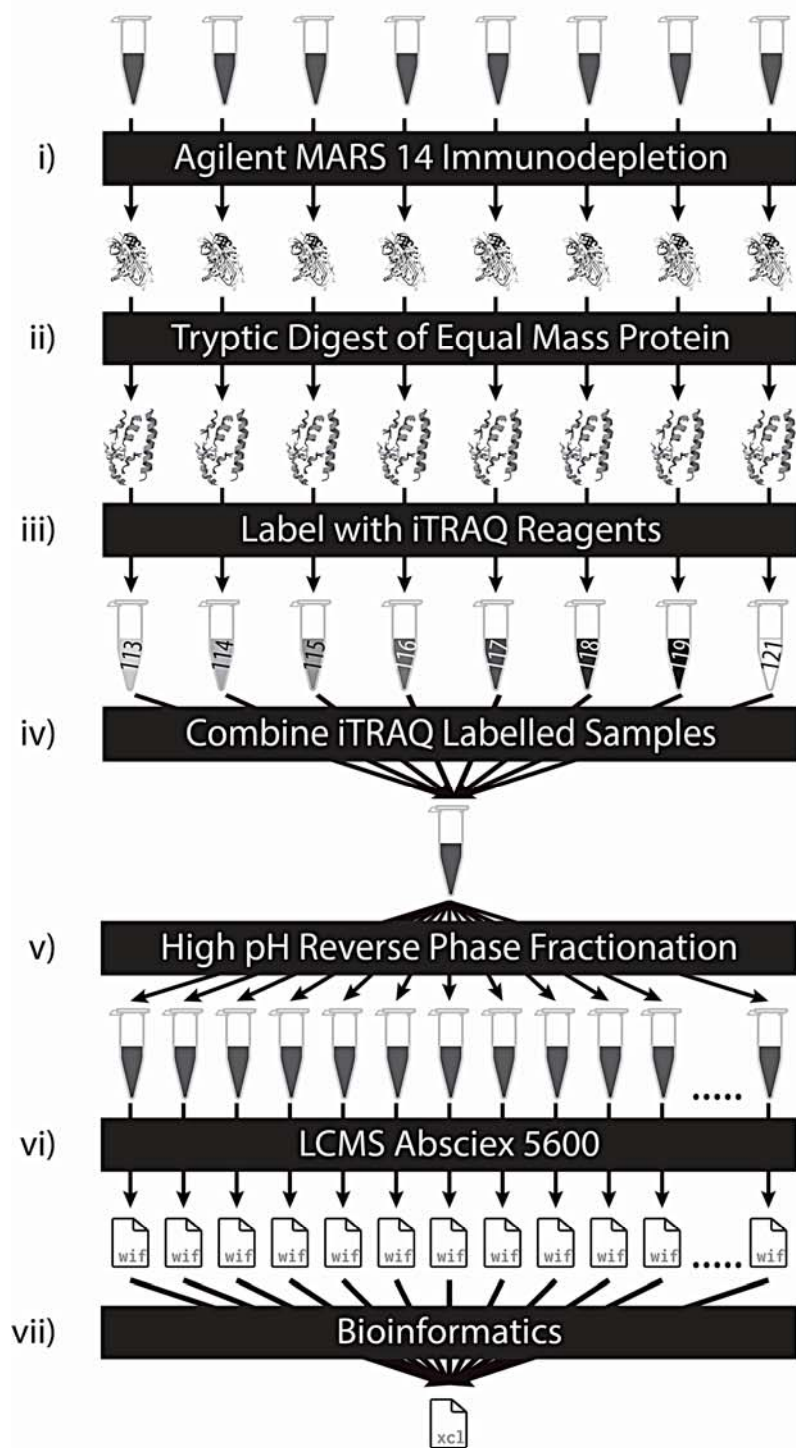

B

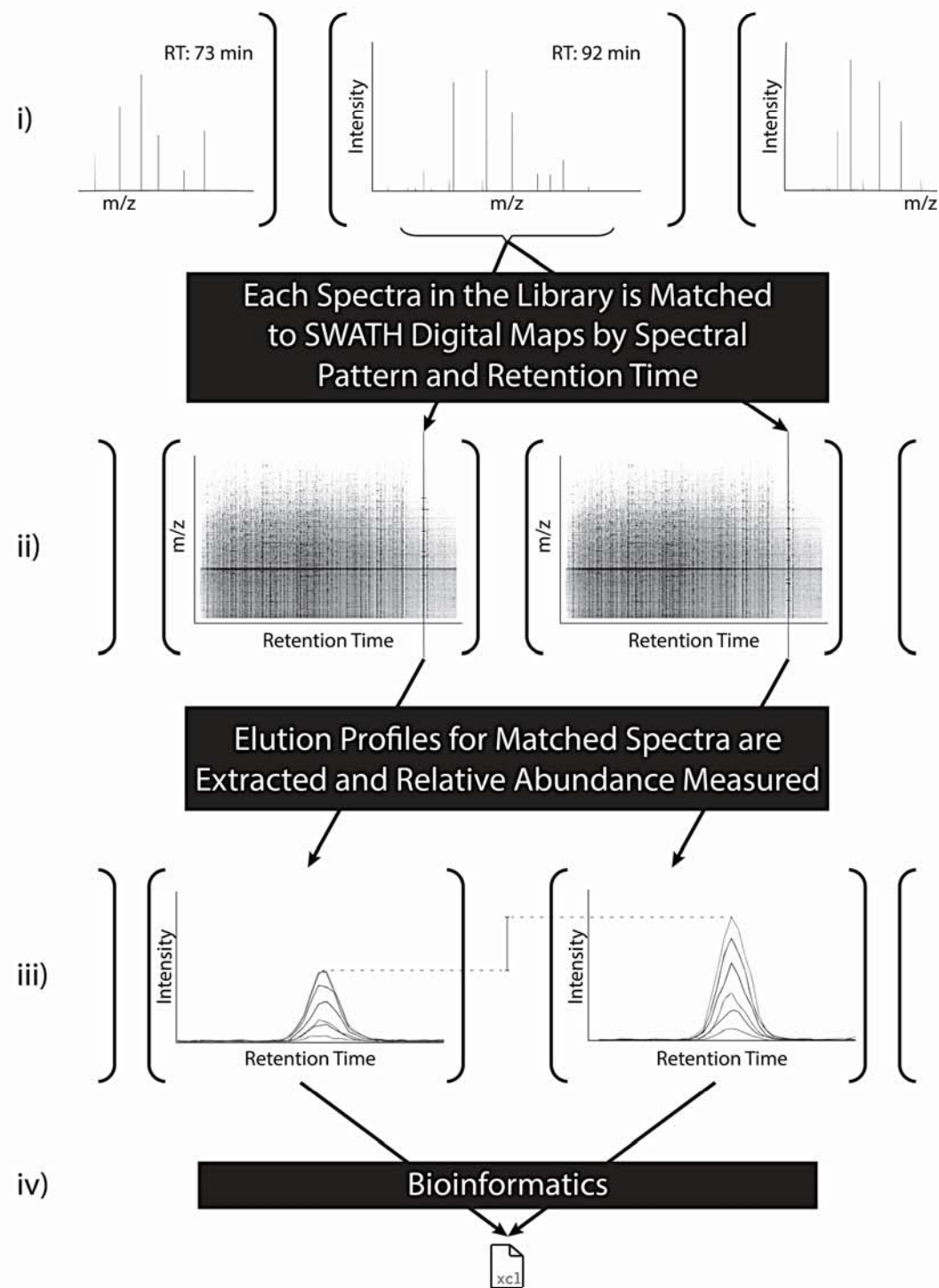

Supplement: Supplementary file 2 — Supporting Information Figure 1 [file IJC-138-2984-s002.pdf]

I)

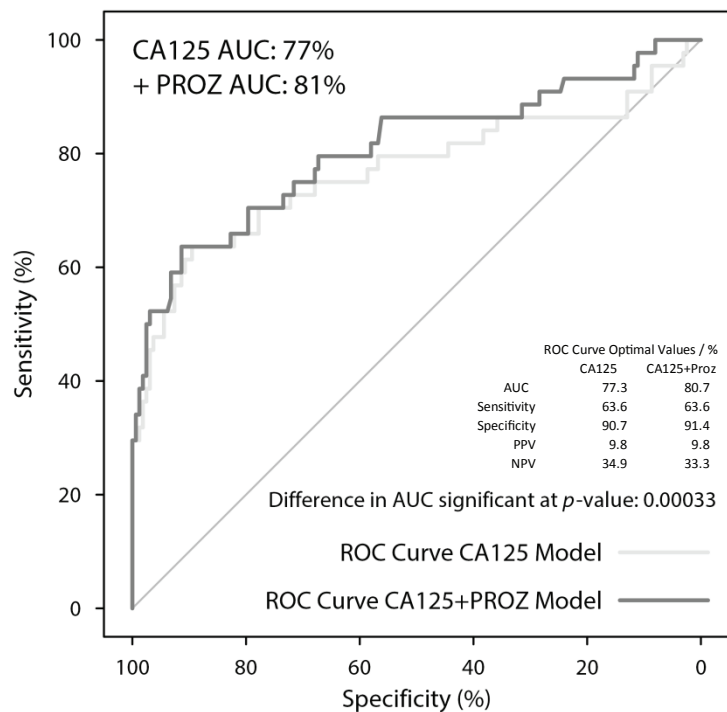

II)

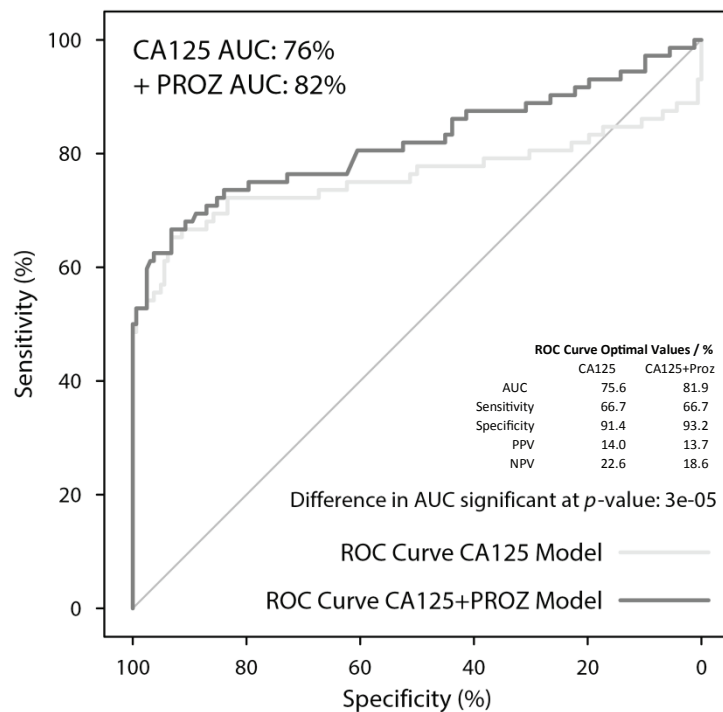

III)

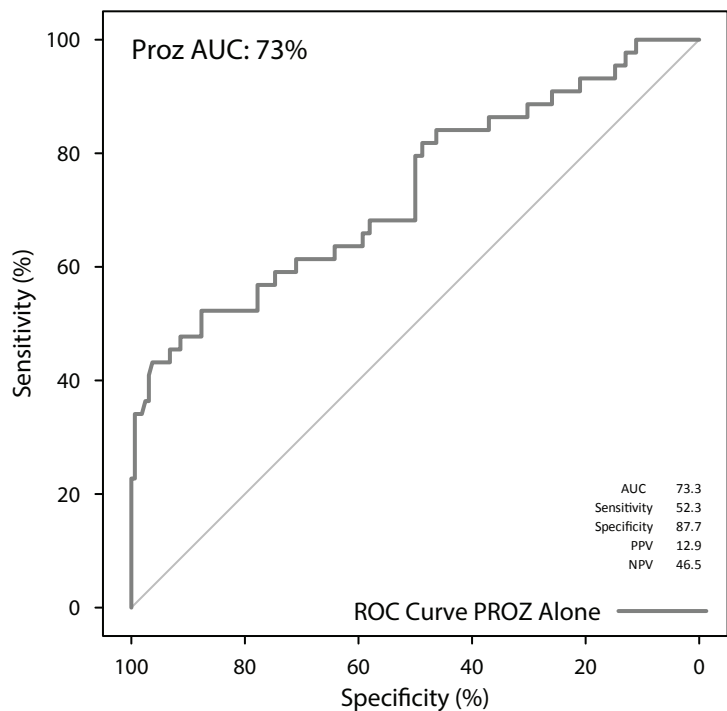

IV)

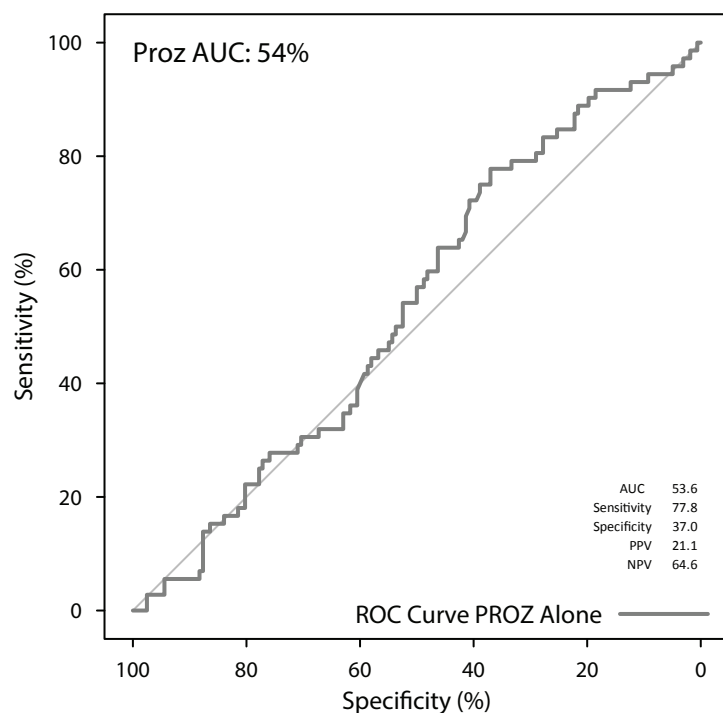

Supplement: Supplementary file 3 — Supporting Information Figure 2 [file IJC-138-2984-s003.pdf]

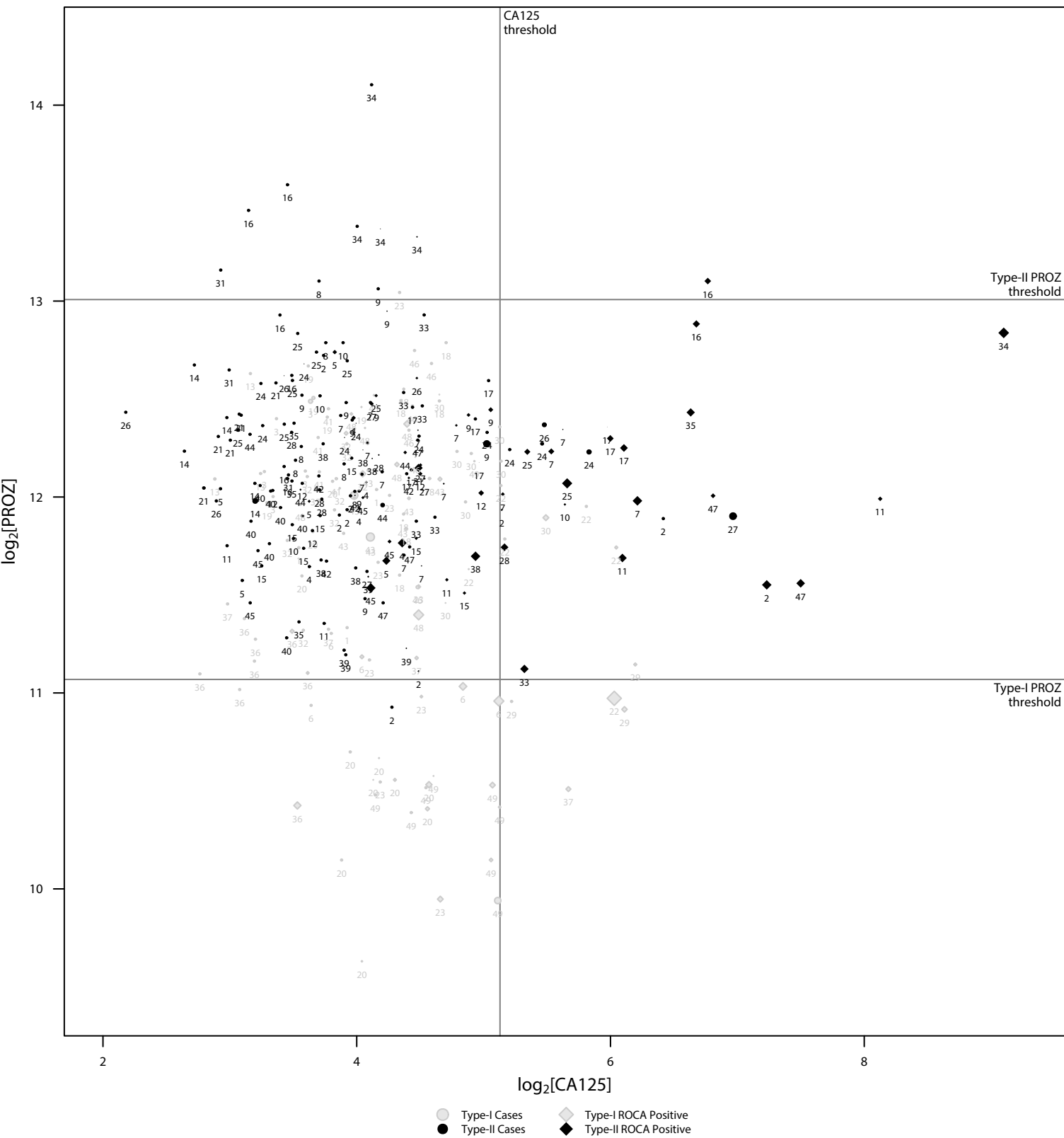

Supplement: Supplementary file 4 — Supporting Information Figure 3 [file IJC-138-2984-s004.pdf]
