# Supplementary material for: Protein Z: A putative novel biomarker for early detection of ovarian cancer
Source: Int J Cancer. 2016 Feb 19;138(12):2984–92. doi: 10.1002/ijc.30020 (PMC4840324; doi:10.1002/ijc.30020)
Supplement: Supplementary file 6 — Supporting Information Table 2 [file IJC-138-2984-s006.docx]

| Pool | iTRAQ Label | No. of Samples | Study Arm | Time to Diagnosis / days | BMI  / kg m^­‑2^ | Age at Sample  / years |
| --- | --- | --- | --- | --- | --- | --- |
| Control <14 months pseudo tDx | 117 | 25 | Control | 154 (79) | 25 (3) | 66.5 (5) |
| Control >32 months pseudo tDx | 115 | 25 | Control | 1762 (396) | 25 (3) | 62.1 (5) |
| Type-I <14 months tDx | 116 | 25 | Type-I | 210 (117) | 27 (5) | 68.8 (7) |
| Type-I >32 months tDx | 114 | 25 | Type-I | 1745 (515) | 27 (5) | 64.6 (6) |
| Type-II <14 months tDx | 118 | 25 | Type-II | 154 (79) | 27 (5) | 66.8 (5) |
| Type-II >32 months tDx | 113 | 25 | Type-II | 1824 (547) | 27 (5) | 62.2 (5) |
