# Supplementary material for: Protein Z: A putative novel biomarker for early detection of ovarian cancer
Source: Int J Cancer. 2016 Feb 19;138(12):2984–92. doi: 10.1002/ijc.30020 (PMC4840324; doi:10.1002/ijc.30020)
Supplement: Supplementary file 7 — Supporting Information Table 3 [file IJC-138-2984-s007.docx]

| Pool | No. of Samples | Study Arm | Time to Diagnosis / days | BMI  / kg m^­‑2^ | Age at Sample  / years |
| --- | --- | --- | --- | --- | --- |
| Control <14 months pseudo tDx | 23 | Control | 158 (82) | 25 (3) | 66.1 (5) |
| Control >32 months pseudo tDx | 23 | Control | 1754 (407) | 25 (3) | 61.7 (5) |
| Type-I <14 months tDx | 19 | Type-I | 203 (106) | 27 (5) | 69.1 (7) |
| Type-I >32 months tDx | 19 | Type-I | 1702 (525) | 27 (5) | 65.0 (6) |
| Type-II <14 months tDx | 27 | Type-II | 147 (74) | 27 (5) | 66.7 (5) |
| Type-II >32 months tDx | 27 | Type-II | 1797 (545) | 27 (5) | 62.2 (5) |
