# Supplementary material for: Protein Z: A putative novel biomarker for early detection of ovarian cancer
Source: Int J Cancer. 2016 Feb 19;138(12):2984–92. doi: 10.1002/ijc.30020 (PMC4840324; doi:10.1002/ijc.30020)
Supplement: Supplementary file 9 — Supporting Information Table 5 [file IJC-138-2984-s009.doc]

| **Individual** | **Histology** | **Morphology** | **Stage** | **Grade** | **Time to Diagnosis / days** |
| --- | --- | --- | --- | --- | --- |
| 1 | Primary borderline epithelial malignant neoplasm | Serous cystadenoma, borderline malignancy (C56) | Ic | - | 1066; 136 |
| 2 | Primary invasive epithelial malignant neoplasm | Endometriod carcinoma (C56) | Ic | Grade 2 | 989; 128; 98 |
| 3 | Primary borderline epithelial malignant neoplasm | Endometrioid adenoma, borderline malignancy (D39.1) | Ia | - | 1820; 1098; 979; 722; 575; 335; 238; 156 |
| 4 | Primary borderline epithelial malignant neoplasm | Endometriod adenofibroma, borderline malignancy (D39.1) | Ia | - | 1951; 1561; 1477; 684; 391; 278; 174; 125 |
| 5 | Primary borderline epithelial malignant neoplasm | Serous cystadenoma, borderline malignancy (C56) | Ia | - | 2394; 380; 170 |
| 6 | Primary invasive epithelial malignant neoplasm | Endometriod adenofibroma, malignant (C56) | IIa | Grade 2 | 1063; 181 |
| 7 | Primary invasive epithelial malignant neoplasm | Clear cell adenocarcinoma NOS | Ia | High Grade | 200 |
| 8 | Primary borderline epithelial malignant neoplasm | Serous cystadenoma, borderline malignancy (C56) | Ib | Borderline | 69 |
