# Supplementary material for: Protein Z: A putative novel biomarker for early detection of ovarian cancer
Source: Int J Cancer. 2016 Feb 19;138(12):2984–92. doi: 10.1002/ijc.30020 (PMC4840324; doi:10.1002/ijc.30020)
Supplement: Supplementary file 10 — Supporting Information Table 6 [file IJC-138-2984-s010.doc]

|  | **Individual** | **lead time over ROCA / days** |
| --- | --- | --- |
| Type-I | 1 | 714 |
|  | 2 | 1484 |
|  | 3 | 737 |
|  | 4 | 2014 |
|  | 5 | 1951 |
| Type-II | 1 | 1096 |
|  | 2 | 2252 |
|  | 3 | 1601 |
|  | 4 | 730 |
|  | 5 | 286 |
