# Supplementary material for: Protein Z: A putative novel biomarker for early detection of ovarian cancer
Source: Int J Cancer. 2016 Feb 19;138(12):2984–92. doi: 10.1002/ijc.30020 (PMC4840324; doi:10.1002/ijc.30020)
Supplement: Supplementary file 11 — Supporting Information Table 7 [file IJC-138-2984-s011.doc]

| **Individual** | **Histology** | **Morphology** | **Stage** | **Grade** | **Time to Diagnosis / days** |
| --- | --- | --- | --- | --- | --- |
| 1 | Primary invasive epithelial malignant neoplasm | Papillary serous cystadenocarcinoma (C56) | IIIc | Grade 3 | 1689; 1337; 959; 507 |
| 2 | Primary invasive epithelial malignant neoplasm | Serous cystadenocarcinoma NOS (C56) | IIIc | High Grade | 2392; 1244; 140 |
| 3 | Primary invasive epithelial malignant neoplasm | Serous cystadenocarcinoma NOS (C56) | Ic | High Grade | 1209 |
| 4 | Primary invasive epithelial malignant neoplasm | Serous cystadenocarcinoma NOS (C56) | IIa | High Grade | 784 |
| 5 | Primary invasive epithelial malignant neoplasm | Serous cystadenocarcinoma NOS (C56) | Ic | Grade 3 | 526 |
|  |  |  |  |  |  |
